# Adolescent Smoking in Secondary Schools that Have Implemented Smoke-Free Policies: In-Depth Exploration of Shared Smoking Patterns

**DOI:** 10.3390/ijerph16122100

**Published:** 2019-06-13

**Authors:** Michael Schreuders, Loekie Klompmaker, Bas van den Putte, Anton E Kunst

**Affiliations:** 1 Department of Public Health, Amsterdam Public Health Institute, Amsterdam UMC, University of Amsterdam, 1105 AZ Amsterdam, The Netherlands; L.Klompmaker@gmail.com (L.K.); A.Kunst@amc.nl (A.E.K.); 2 Faculty of Social and Behavioural Sciences, Department of Communication, University of Amsterdam, 1018 WV Amsterdam, The Netherlands; S.J.H.M.vandenPutte@uva.nl

**Keywords:** Smoking, adolescents, smoke-free school policies, tobacco control, shared smoking patterns, social context

## Abstract

Large numbers of adolescents smoke during school hours, despite the implementation of smoke-free school policies (SFSPs). Studies about SFSPs predominantly analyse smoking as individual behaviour, yet there is increasing recognition that smoking should be understood as social behaviour. We explored shared smoking patterns specifying *where*, *when*, and *with whom*, and social meanings about *why* groups of adolescents smoke in two Dutch schools that have implemented SFSPs. Surveys among adolescents were held to obtain contextual information about the schools. Four focus group discussions and fourteen individual interviews were held with adolescents to identify shared smoking patterns in each school. Two shared patterns were identified at a school where 17% of students smoked daily: Dependent smoking and Rebellious smoking. Both built on pro-smoking norms and underscored the benefits of smoking. Three shared patterns were identified at a school where 3% of students smoked daily: Social bonding smoking, Low-profile smoking and Smoking-friendly event smoking. These built on anti-smoking norms and helped smokers cope with negative social judgements related to smoking. We conclude that adolescent smoking during school hours is embedded in diverse shared smoking patterns. Future studies should develop more understanding about how to deal with adolescents’ shared smoking patterns that decrease the effectiveness of tobacco policies.

## 1. Introduction

Policy makers in many European countries have adopted legislation that compels secondary schools to implement smoke-free school policies (SFSPs). The aim of SFSPs is to limit tobacco use by defining who is prohibited to smoke where and when, and prescribing what consequences will follow when rules are violated. Political support for SFSPs can be explained by evidence about the high proportion of smokers that have taken up smoking during adolescence [1], the influence of the secondary school environment on adolescents’ smoking uptake and continuation [2] and the widespread support for smoke-free measures [3,4].

Contemporary studies assessing the impact of SFSPs on adolescent smoking behaviour present largely inconsistent results [5]. A key reason for this inconsistency could be that studies hardly take into account that schools involve “numerous, dynamic, highly interactive, learning and adaptive” individuals who “act in ways that are based on a combination of their knowledge, experience, feedback from the environment, local values and formal system rules” [6]. This complex systems perspective on schools has two implications for understanding the impact of SFSPs. First, the implementation of SFSPs may elicit different responses among different adolescents. These different responses can, among others, be influenced by differences in school characteristics (e.g., levels of SFSP implementation and smoking prevalence) and individual characteristics (e.g., educational level and exposure to family smoking). Second, adolescents’ responses to SFSPs may elicit subsequent responses among other adolescents. Schools involve multiple—not necessarily well-defined—groups of adolescents that may use (non-)smoking behaviours as a means to distinguish themselves from others. SFSPs thus likely have a different impact among schools and among groups of adolescents within the same school.

Building on these implications, a recently published literature review identifies adolescents’ cognitive and behavioural responses to the implementation of SFSPs [7]. One key response is that SFSPs may reduce the pressure to conform to others’ smoking behaviours. This response parallels the commonly held view that smoke-free environments denormalise the use of tobacco: starting and continuing smoking is not cool anymore [8], but rather, it is a deviation from the societal norm to abstain from smoking [9,10]. However, SFSPs may concurrently lead to the development of social meanings that increase some adolescents’ experience of pressure to smoke [7]. These pro-smoking social meanings denote the positive social expectations and social significance that some adolescents attach to smoking, and that may consequently lead them to initiate and perpetuate smoking [11,12]. Two empirical studies showed that some adolescents see smoking as a means to rebel against the school rules on smoking [13,14].

The pro-smoking social meanings in schools that have implemented SFSPs can best be understood by analysing smoking not as an individual behaviour, but instead as an element in adolescents’ “shared way of relating and acting in a given environment” [15]. Adolescent smokers within a single school likely belong to a group that manifests a shared way of relating and acting during school hours; groups of smoking adolescents may distinguish themselves from other smoking groups by following different, what we will call, “shared smoking patterns” that specify *where*, *when*, and *with whom*, and social meanings about *why* they smoke during school hours. These shared smoking patterns may contribute to adolescents smoking persisting during school hours by allowing smokers to collectively adapt their smoking to deal with stronger SFSPs and by attracting non-smokers to follow similar patterns. [11,12].

To our knowledge, no scientific literature exists that has studied adolescents’ shared smoking patterns in schools that have implemented SFSPs. Studies on SFSPs build predominantly on social psychological theories, and consequently, focus on individual-level responses, and not so often on social-level responses [7]. Scholars, however, increasingly argue that understanding social-level responses to tobacco control policies is key for further reducing adolescent smoking, particularly among those with a lower socio-economic status [16]—that is, adolescents who come from backgrounds that benefitted the least from contemporary tobacco control measures [17]. We therefore performed this small-scale cross-sectional study at one low-level school (i.e., vocational) and one mid-level school (i.e., theoretical) in the Netherlands, aiming to explore whether adolescents show shared smoking patterns during school hours in secondary schools that have implemented SFSPs and, if so, what are the shared smoking patterns in these two schools.

## 2. Materials and Methods

This study is part of the European SILNE-R project (2015–2018) that aims to develop insights for enhancing the impact of common tobacco control measures on youth smoking (http://silne-r.ensp.org/). We used survey and focus group discussion data that was collected during the school year 2016–2017 for the SILNE-R project, from 3rd and 4th grade students (i.e., predominantly 14–17 years of age in Dutch educational system) at two secondary schools in Amersfoort. After an initial analysis quickly following SILNE-R data collection, we performed additional interviews with students at these schools during the same school year. We also used staff interview data that was collected in the same period for the SILNE-R project to get an overview of each school’s SFSP. This did not involve a substantive analysis, and therefore, will not be discussed separately.

The low-level school was called “Highsmoke” and the mid-level school was called “Lowsmoke”, based on their relatively high and low smoking prevalence (see Results). The schools were located only 500 m apart and had prohibited smoking on the school premises for at least three years at the time of data collection. Legislation prohibiting any smoking at the school premises comes only into effect by 2020, yet many schools have voluntarily implemented some form of prohibition.

### 2.1. Survey

The SILNE-R survey was used to provide contextual information about the gender distribution, weekly smoking prevalence, and proportion of adolescents exposed to family smoking per school. Information about the procedure is reported elsewhere [18].

### 2.2. Focus Group Discussions

The SILNE-R focus group discussions (FGD) transcripts were used to identify the shared smoking patterns at each of the schools. Twenty-two students aged 14–17 participated in two single-gender FGD per school. Single-gender groups were chosen because the reasons for and experiences of smoking may vastly differ between genders [19]. Recruitment of adolescents was done in close collaboration with teachers. Adolescents were purposefully sampled and eligible for recruitment if they were either smokers or if teachers considered them to be susceptible to starting smoking (e.g., smoking parents or friends).

The FGD followed a semi-structured guide. The FGD were held in February and March 2017, lasted 45–60 min, and took place in a private and quiet room in the school during the school hours. All FGD were facilitated by a male PhD-candidate (lead facilitator and first author of this paper) and a female PhD-candidate (supportive role), whom both had training in qualitative data collection and analysis. Participants received no reimbursement for participation.

The facilitator explained the purpose of the FGD and adolescents’ rights as confidential participants in scientific research, and asked them for their written consent and approval for voice-recording the FGD. The FGD explored a multitude of topics that were mostly beyond the scope of this study on shared smoking patterns. In our analysis below, we concentrate on discussions among adolescents about their perceptions of smoking during school hours and smoke-free school policies.

### 2.3. Analysis of FGD Transcripts

The FGD were voice-recorded and transcribed verbatim. MaxQDA12 (VERBI GmbH, Berlin, Germany) was used for electronically sorting the data and the coding.

We applied a framework analysis [20]. The coding framework built on the expectation that groups of adolescents will show similar patterns in *where*, *when*, *with whom* and *why* adolescents do or do not smoke during school hours. After thoroughly reading the FGD transcripts, the first author started the analysis by coding any part of the text that helped to develop a view of adolescent smoking during school hours. Then, a subsequent analysis to find themes within these coded texts resulted in initial patterns explaining where, when, with whom and why groups of adolescents do or do not smoke during school hours. A pattern meant there was a certain way of smoking (i.e., at the same place(s) and time(s), with the same adolescents and for the same reason(s)) that was recognized by and shared among multiple adolescents within a school, and that could be distinguished from other ways of smoking. Most codes that came up during the FGD analysis involved the smoking of students near the school area. We chose to further explore the patterns in adolescents’ smoking near the school area by performing additional interviews. Interviews were preferred, as these allowed us to delve more deeply in adolescents’ personal accounts of the shared smoking patterns.

### 2.4. Interviews

The analysis of FGD provided us insights about *where* smoking during the school hours occurs and the first author went to the schools to observe if these were indeed the main locations where adolescents smoke. The first and second authors later purposefully went to these different locations during the school hours to recruit adolescent smokers for participation in the interviews.

Fourteen students aged 15–18 participated in individual interviews. The second author (female) performed all interviews as part of her training in qualitative research methods; these interviews were repeatedly discussed with the first and last authors. Heterogeneity of participants was realized by aiming to include an equal number of boys and girls, resulting in four girls and three boys per school. Three participants had also participated in the FGD.

The interviews followed a semi-structured interview guide to probe further on where, when, with whom and why the interviewee did or did not smoke during school hours. Earlier acquired insights from the FGD were used for probing. The interviews were held in April and May 2017, lasted about 30 min and took place in a private and quiet room in or near the school. All interviewees received €10 for participation because the interviews did not take place during school hours.

The second author performed the interviews. She first explained the purpose of the interview and adolescents’ rights as confidential participants in scientific research, and asked them for their written consent and approval for voice-recording the interview. The interview started by asking adolescents about the smoking rules that apply at their school. The interview was then navigated to adolescents’ own experiences with smoking and where they smoke during school hours. Talking about where they smoke was a good starting point to explore when, with whom and why they smoke at these places during school hours. The interviewer concurrently probed the adolescent smokers to elaborate upon where, when, with whom and why they do not smoke during school hours, as this contrasting assisted adolescents in providing in-depth insights into what distinguishes the different shared smoking patterns within school.

### 2.5. Analysis of Interview Transcripts

The interviews were voice-recorded and transcribed verbatim. MaxQDA12 was used for electronically sorting the data and the coding.

The analysis of the interviews built further on what was found during the analysis of FGD and hence focused on enriching and triangulating the patterns explaining where, when, with whom and why groups of adolescents do or do not smoke during school hours. The first and second author read all transcripts. The first and second author coded the transcripts and met several times to discuss and find consensus. Meetings with the other authors were held for discussing the coding. All authors agreed with the shared smoking patterns presented in the Results section.

### 2.6. Ethics

The issue of ethics was discussed with the schools, and together, we agreed on a protocol, including the informing of parents. The protocol was sent to the Amsterdam Medical Centre Medical Ethics Review Committee, which concluded that the Medical Research Involving Human Subjects act does not apply to this study and that therefore further approval was not required. All participants were informed about the study, their right of participants in scientific research and asked for written approval.

## 3. Results

The results for Highsmoke (H) and Lowsmoke (L) are presented separately. The codes related to the quotes below refer to the participants. The first letter refers to their school (H or L). The second letter refers to male (M) or female (F). The third character is D if it was said in a FGD or a number if it was said in an interview.

### 3.1. Highsmoke

Highsmoke was a vocational school with 250 adolescents. Adolescents did not attend school five days a week, but had a work placement for up to four days per week. The survey showed that 49% of 3rd and 4th graders had ever smoked and that 17% smoked daily. Also, 46% were girls and 50% were exposed to at least one smoking parent.

Highsmoke prohibited smoking in the school buildings and on the premises. However, adolescents were allowed to smoke in a designated smoking area outside the premises, but that was accessible only via the premises and located adjacent to the area where non-smokers spend their breaks. Non-smokers could stand on the school premises near to the smokers. Some areas outside the school premises were also used for smoking, which provided some basic facilities, including shelter and a bench. Highsmoke allowed adolescents from the 4th grade onwards to smoke. Highsmoke actively prevented the school timetables from having free-hours in between lessons. This technically meant that smoking was limited to 4th graders onwards and during breaks.

Adolescents at Highsmoke perceived smoking as normal behaviour; smokers easily admitted their nicotine addiction, did not feel ashamed to smoke alone or in the full sight of others, and did not feel judged for smoking by others. We identified two shared smoking patterns that underscored the benefits of smoking during school hours: Dependent smoking and Rebellious smoking.

#### 3.1.1. Dependent Smoking

- Where: official smoking area;
- When: every school break;
- With whom: anyone who wants to smoke or stand near a smoker;
- Why (social meaning): deal with stress and need for nicotine.

The majority of smokers smoked during school breaks at the school’s official smoking area. School breaks were seen as an opportunity to consume the necessary amount of nicotine to get through the upcoming hours, as there were no other opportunities to smoke.

Moderator: “How much do you smoke during a break?”

Girl1: “Two”.

Girl2: “Me too”.

Moderator: “So what if you’d smoke only one?”.

Girl1: “Then I go crazy (…) can’t sit still, no more concentration and can only think of that [smoking]” (HFD).

Boys and girls smoked together in an indistinct group that also included non-smokers. They stood either alone, with their friends or with anyone who is having a smoke. Smoking during school hours was widely accepted; most adolescents did not judge smokers as those against smoking were clearly framed as the minority.

“I don’t think so because there are many smokers (…) The only ones who really are against smokers are those ‘real anti-smokers’” (HF1).

The widespread acceptance of smoking related to adolescents’ perceptions that everyone could be a smoker and that there are sensible external reasons that could cause someone to start smoking and so become nicotine dependent.

Boy1: “When I joined this school I noticed right away that there is a lot of smoking”.

Boy2: “I think about a quarter of the school”.

Boy1: “Maybe even half”.

Boy3: “Even those individuals you don’t expect”.

Boy1: “Yes, these correct types.” (HMD).

One reason they gave for starting smoking was that it reduces stress. This stress was oftentimes associated to the school setting or the home environment. Another reason was group pressure. The group pressure at school was quite strong, as some adolescents stated that they need to actively prevent themselves from giving in to this group pressure.

“I protect myself by not standing there [near smokers] (…) I’m afraid that they will ask me [to smoke] and I’m not the type of person that easily says no” (HMD).

The decision to start smoking was nevertheless generally seen as an individual choice because someone always has the option to say no. Both smokers and non-smokers therefore argued that adolescents who *need* to smoke during school hours should not be withheld from the opportunity to smoke a cigarette.

Girl1: “It is their own life and if they want to smoke because they really need it, then it should be possible” (HFD).

#### 3.1.2. Rebellious Smoking

- Where: outside the school premises;
- When: every school break;
- With whom: single-gender friend groups;
- Why (social meaning): expresses toughness.

A minority of smokers smoked during school breaks at locations just outside the school premises, predominantly behind a bus station with an adjacent bench. These adolescents smoked in single-gender friend groups that consisted only of smokers. They smoked outside the premises because it allowed them to meet with adolescents from other schools and it gave them privacy from teachers’ supervision.

“People from other schools come to us, but they’re not allowed to enter the school premises, so we stand there [behind a bus station]” (HF2).

Boys who smoked near the bus station described the girls smoking there as “feeling unashamed” and “standing with their legs far apart [non-feminine]” (HM1) and framed smokers outside the premises as “tougher” than the others. They underscored this toughness by talking about their minor violations of the smoking rules that teachers did not sanction.

“Rules exist to be broken (…) I light a cigarette and when the bell rings and I’m not finished yet, then I walk over the [smoke-free] school premises with my cigarette” (HM1).

Other adolescents, including those who smoked only in the official smoking area, judged harshly about the boys and girls smoking outside the school premises. Individuals were described as offensive and it was generally believed that they try to “act tough in front of teachers” (HF3).

Boy1: “Simply these unmannered children”.

Boy2: “Those of the street”.

Boy3: “Scum”.

Boy2: “Yes, street scum” (HMD).

These perceptions made most smokers decide not to smoke outside the school premises, reasoning that these smokers are different and do not want to be associated with them.

“No, I don’t smoke there. There’s always these people. The tougher people” (HF4).

### 3.2. Lowsmoke

Lowsmoke was a school with 750 adolescents that provided full-time mid-level education. The survey showed that 29% of 3rd and 4th graders ever smoked and 3% smoked daily. Also, 52% were girls and 27% were exposed to at least one smoking parent.

Lowsmoke prohibited smoking in the school buildings and on its premises. The only area where adolescents were allowed to smoke was just outside the entrance, which everyone referred to as *the gate*. Smokers were only allowed to smoke at this area because Lowsmoke did not want smoking to be comfortable; there was no bench to sit or roof to shelter for rain. There was a parking lot between *the gate* and the school premises, physically separating smokers from non-smokers. Lowsmoke only allowed the 3rd and 4th graders to leave school premises during school hours, including free time between teaching hours that occurred due to gaps in adolescents’ timetables. This technically meant that only 3rd graders onwards were allowed to smoke during breaks and free-hours.

Adolescents at Lowsmoke perceived smoking as a norm-breaking behaviour; smokers did not feel completely comfortable smoking, and felt judged by others. We identified three shared smoking patterns that helped smokers cope with the negative judgements associated with smoking during school hours: Social bonding smoking, Low-profile smoking and Smoking-friendly event smoking.

#### 3.2.1. Social Bonding Smoking

- Where: just outside the school premises;
- When: every school break;
- With whom: daily smoking boys who isolate themselves as a group from non-smokers;
- Why (social meaning): daily smoking is an indispensable part of their membership to a group that creates a smoking-tolerant environment.

A group of boys smoked at the gate during all school breaks. These boys were slightly older than other adolescents and explicitly positioned their group as separate from the rest; “[we] do not really engage with anyone else at school” and “absolutely do not care about what others think [of us]” (LMD). This group was difficult to join for outsiders, non-smokers in particular.

“Since a few years we have a group of friends that spend the school breaks together. A few months ago he [another student] suddenly joined us and now stands with us every time. He thinks he is part of us, but he never smokes. We all say to him ‘why are you standing here, you don’t even smoke?’. We don’t have friends standing with us that do not smoke” (LM2).

Smoking created a group vibe that does not exist without smoking. They talked about this vibe by referring to “good memories” (LM1) when they were smoking together and how these thoughts made them want to smoke in social situations.

They smoked together during school breaks because it had become a behavioural routine that was difficult to stop. Spending school breaks without smoking was not even considered an option, as they found it difficult not to smoke in the presence of their friends and were not closely befriended with most non-smokers.

Boy1: “If you chose not to smoke, you’ll be alone [without friends] in the school building as you do not know anyone.”.

Moderator: “But you could stand at the gate without smoking?”.

Boy1: “Yes that’s true, but you know.”.

Boy2: “Then you’re offered a cigarette.”.

Boy1: “That’s the danger” (LMD).

Boys smoking during free time between teaching hours was not as common as smoking during school breaks because they had different free periods due to individualized school timetables, depending on which courses they take. Smoking during school breaks was different as “you do it with everyone and so don’t have to feel alone”. (LM3) Smoking with others allowed them to hide in the crowd and therefore feel “less looked at” (LM1) like addicts.

Boy: “I don’t do that [smoking during free time between teaching hours]. Then you have to stand alone at the gate. That looks strange, really like you’re a junkie.”

Interviewer: “Why do you think so?“.

Boy: “It looks like you really cannot live without. That looks so horrible” (LM3).

Smoking almost exclusively with close friends was their way of creating a small smoking-tolerant environment in a larger smoking-intolerant school context.

#### 3.2.2. Low-Profile Smoking

- Where: first behind the flats, but since recently just outside school premises;
- When: when they occasionally feel like;
- With whom: girls who stand outside with their (non-)smoking friends;
- Why (social meaning): smoking occasionally and only for pleasure prevents others from thinking that they are addicted or smoke to impress.

There were a few groups of girls mixed of smokers and non-smokers. Smoking was not a prerequisite for group membership; “if one of the girls smokes, the group stands outside [school premises].” (LF1) Most girls did not want to smoke every school break or free hour and only smoked when they would enjoy it. Smoking was, for instance, strongly connected to enjoying being outside, “particularly when the weather is nice.” (LF3)

“Not all of us smoke every day. You only smoke when you feel like it.” (LF2).

“I smoke only sometimes at school. I don’t have these urges where I think ‘now I need a cigarette’” (LF4).

These girls underpinned that they did not want to smoke every school break by explicitly distancing themselves from the boys that go out to smoke every school break.

Girl: “Sometimes I see the boys of our school who smoke on a regular basis. They have so much desire [for a cigarette] and when I see it, I think ‘I don’t want to be like that” (LFD).

Formerly, the girls used to walk to nearby flats as they “felt ashamed to smoke near the school gate” (LFD). These flats allowed them to smoke covertly so that fellow students and individuals passing the school would not notice them. School, however, recently prohibited them from smoking near these flats. The girls reasoned that school “purposefully decided that we are not allowed to smoke near the flats (…) they know we feel ashamed to smoke at the gate, so they think we will stop smoking” (LFD). They didn’t stop smoking and instead started smoking occasionally at the gate. The girls explicitly stressed that they, in contrast to others, don’t smoke at the gate to look cool and impress, but only smoke for authentic reasons, like for pleasure.

“You smoke for yourself, not so that everyone can see it, right?“ (LF4).

#### 3.2.3. Smoking-Friendly Event Smoking

- Where: socio-spatial environments where smoking is accepted;
- When: after school hours;
- With whom: anyone who is present;
- Why (social meaning): feel free to smoke without risking negative social consequences.

Some adolescent smokers refrained from smoking during school hours. They were afraid that fellow students will judge them harshly if they would find out that they smoke. They preferred to smoke during smoking-friendly events as they do not have to worry about the possibility that someone will negatively judge them for smoking.

“I have friends who say they don’t smoke at school, but smoke during parties or when we sit on a terrace. They don’t feel comfortable to smoke at school” (LF4).

This shared smoking pattern was predominantly attributed to girls because their smoking during school hours was easily judged as wanting to look cool and trying to impress.

“People will think badly about you [as a girl] and say that you smoke to look cool” (LF1).

These girls therefore wanted “as little people as possible” (LF2) to see that they smoke, so that “not everybody knows about it” (LF3). Boys similarly mentioned that primarily girls experience such concerns, likely because they themselves argued not to care so much about other adolescents’ opinions.

“She has a lot of friends at school. She doesn’t want to portray a picture of herself like ‘look at me’. So she smokes, but definitely not at school.” (LM3).

Adolescents who smoked during school hours also recognized these concerns, and therefore, preferred to smoke at social events where smoking is more accepted. Such environments allowed them to smoke without having to think of others’ opinions.

“It is not that I constantly think ‘shit, these people are looking at me’ (…) but I just prefer to smoke on parties or whatever, because then it is more accepted” (LM2).

## 4. Discussion

This study aimed to develop a better understanding about how adolescents’ smoking during school hours persists in schools that have implemented SFSPs, by exploring whether adolescents in these schools show shared smoking patterns and, if so, what are the shared smoking pattern in these schools.

Five shared smoking patterns were identified. Two shared smoking patterns were identified at Highsmoke. ‘Dependent smoking’ (smoking in a large group at the official smoking area helps to deal with stress and nicotine addiction) and ‘Rebellious smoking’ (smoking in friendship groups outside the school premises expresses toughness). Three shared smoking patterns were identified at Lowsmoke. ‘Social bonding smoker’ (boys’ daily smoking outside the premises in an exclusive smokers’ group creates a smoking-tolerant environment), ‘Low-profile smoking’ (girls’ occasional smoking outside the premises for personal pleasure prevents others from thinking they are addicted or smoke to impress) and ‘Smoking-friendly event smoker’ (smoking only after school hours at smoking-friendly events makes them feel free to smoke without risking negative social judgements).

### 4.1. Limitations

A first limitation is that we collected data only at one point in time, and therefore, could not unravel the causal influence of SFSPs on the shared smoking patterns. The shared smoking patterns may have existed before SFSPs were implemented, and differences in SFSPs and shared smoking patterns could be related to differences in educational level and the smoking prevalence between both schools.

A second limitation is that we did not take into account the socio-demographic characteristics of adolescents who participated in the FGD and interviews. Smokers from Lowsmoke may be intermittent smokers who come from social environments where smoking is frowned upon and smokers at Highsmoke may be heavy smokers who come from environments where smoking is part of daily life. Differences between schools may therefore be an indication of adolescents’ general lifestyle rather than reflecting the school environment.

A third limitation is that we included only two schools. This small number was the result of a collaborative decision made among researchers participating in the SILNE-R project to organise the FGDs only in two or three schools per country. In the Netherlands, FGDs were organized in three schools, yet one school did not allow the collection of additional interview data. Therefore, the generalizability of the precise shared smoking patterns may be limited.

A fourth limitation is that we explored shared smoking patterns only with two FGDs and seven interviews per school. This data collection did not stop upon reaching theoretical data saturation, because we made a priori agreements with schools about the number of FGDs and interviews. We therefore cannot exclude that we missed out shared smoking patterns that are less overt or prevalent.

### 4.2. Interpretation of Findings

Adolescents at Lowsmoke, in contrast to those at Highsmoke, perceived strong anti-smoking norms for smoking during school hours. This manifested in fundamentally different shared smoking patterns. The three shared smoking patterns at Lowsmoke responded to the anti-smoking norms by helping smokers cope with the experience of negative judgements for smoking, whereas the two shared smoking patterns at Highsmoke underscored the benefits of smoking. We will therefore discuss the shared smoking patterns in perspective of smoking denormalization, in particular its role in decreasing smoking [21] and increasing stigma as experienced by smokers [22].

Social bonding smoking at Lowsmoke involved smoking daily as an indispensable part of membership to a group, to create a smoking-tolerant space within a smoking-intolerant environment. This indicates that adolescents may, in response to stigma, form friend groups that almost exclusively consist of and engage with daily smokers, contributing to smokers’ self-induced social isolation from the increasing majority of non-smokers [9]. This corresponds with evidence about the increasing concentration of smokers in smoker-networks [23], and suggests that future tobacco control strategies should not merely target individuals or the population at large, but also the friend group.

Low-profile smoking at Lowsmoke involved smoking occasionally and only for authentic reasons, to prevent others from thinking that one is addicted or smokes to impress. These adolescents recognised the stigma related to smoking, but concurrently argued that good and bad ways of smoking exist. A good way of smoking is, in line with prior literature [24,25], to smoke for yourself and only when you feel like, and bad smoking is to impress or due to nicotine dependence. Those who implicitly categorized themselves as smoking in the good way collectively participated in the stigmatization of the bad smokers. Such downward comparison, also found among Scottish adults [10], is a known strategy for individuals “to agree with negative stereotypes, but resist applying these stereotypes to themselves” [9]. This pattern also corresponds with evidence showing that tobacco control in Western countries has decreased daily smoking, but has failed to decrease occasional smoking [26], signifying the need to address socially reinforced rationalisations that justify occasional smoking.

Smoking-friendly event smoking at Lowsmoke involved smoking only after school hours at smoking-friendly events to avoid risking negative social judgements. Adolescents talked about specific places where and times when smoking is more accepted, and deliberately went to these socio-spatial environments to smoke. Young adults were similarly shown to perceive smoking in bars and cafes in the weekend as settings where the normal expectations about abstaining from smoking do not apply [27]. Scholars also warned that smokers’ increasing perception of stigmatisation may lead to their relocation to so-called smoking islands [28]. These smoking islands may, in turn, increase the persistency of individuals’ smoking because these socio-spatial environments bring together those who resist the anti-smoking norm or feel helpless to quit [29].

Dependent smoking and Rebellious smoking at Highsmoke involved smoking at the designated smoking area to deal with stress and the need for nicotine, or smoking outside the premises to express toughness. These social meanings parallel views that were dominant before smoking became a deviation from societal norms [30], suggesting that anti-smoking campaigns, SFSPs and other tobacco policies may have hardly changed the smoking norms and behaviours of adolescents at some schools.

Despite stark differences in the precise shared smoking patterns, the social dynamics between social in-groups and social out-groups showed commonalities between the two schools. Social bonding smoking (Lowsmoke) and Rebellious smoking (Highsmoke) both violated the norms to such extent that other smokers explicitly distanced themselves from the respective pattern at their school. Also, adolescents following one of these norm breaking patterns in either of the schools described themselves as an ‘exclusive group’ which is difficult to join for the majority of adolescents. On the other hand, Low-profile smoking (Lowsmoke) and Dependent smoking (Highsmoke) were relatively undefined and open for anyone who wants to smoke in a socially acceptable way.

### 4.3. Practical Implications

The strengthening of anti-smoking norms among adolescents is a primary causal mechanism underlying the impact of SFSPs [7]. The results, however, showed that this strengthening of anti-smoking norms may not always happen or have the anticipated impact. First, it seemed that SFSPs are insufficient for strengthening adolescents’ anti-smoking norms in every school. Second, in schools that become more anti-smoking, subsets of adolescents may develop shared smoking patterns that help them to persist smoking in smoking-intolerant school environments. Schools in both cases may benefit from complementary measures to realize further decreases in adolescent smoking behaviour. We will put forward three novel possibilities that need further study. 

First, schools may prohibit smoking during school hours in or outside the premises. The only available study showed that complementing SFSPs with prohibitions from leaving the premises were associated with lower smoking rates compared to other SFSPs [31]. These prohibitions may prevent adolescents from isolating themselves in smoker-only groups (e.g., Social bonding smoking) or schools from becoming smoking islands (e.g., Highsmoke). However, evidence on the impact of this measure is still scarce.

Second, schools may target pro-smoking social meanings that maintain specific forms of adolescent smoking behaviour. Key targets could be occasional smoking during (e.g., Low-profile smoking) and after (e.g., Smoking friendly event smoking) school hours, because occasional smokers have an ambivalent attitude towards smoking. To date, no evidence exists about interventions that aim to address occasional smoking as a social-level phenomenon. Such interventions may be inspired by social design methods [32].

Third, schools may create a physical environment that decreases the chances that adolescents start or continue following shared smoking patterns. A recent study, for instance, suggested that changing the school surrounding, among which creating a pleasant non-smoking area, may reduce progression from occasional to daily smoking [33].

### 4.4. Future Research

This exploratory study may inspire further research on adolescents’ shared smoking patterns and how these contribute to persisting adolescent smoking. Future studies should use more rigorous research designs than ours. For example, Hargreaves et al. [34] studied changes in social-level cigarette consumption patterns in response to tobacco control policies, by collecting data pre-post implementation, using complementary methods, and comparing various localities and settings with different characteristics.

## 5. Conclusions

Schools that have implemented smoke-free school policies are still confronted with adolescents who smoke during school hours. This was shown to be socially embedded in diverse shared smoking patterns that support adolescents in persisting in smoking, despite an increasingly smoking intolerant environment. We therefore suggest new studies that aim to develop more understanding on how to effectively target adolescents’ shared smoking patterns, in an attempt to help further decrease adolescent smoking during and after the school hours.
